# Clinical outcomes of fasting in patients with chronic heart failure with preserved ejection fraction: A prospective analysis

**DOI:** 10.1016/j.amsu.2022.104373

**Published:** 2022-08-18

**Authors:** Shafiq Alam, Shoukat Hussain, Jawad Abbas, Muhammad Hassan Raza, Waqar Arif Rasool, Abdulla K. Alsubai, Razan Al-Mousawi, Khaled Saeed Obaid Aldhaheri, Jahanzeb Malik, Talal Almas

**Affiliations:** Department of Cardiology, Mardan Medical Complex, Mardan, Pakistan; Department of Medicine, Abbas Institute of Medical Sciences, Muzzafrabad, Pakistan; Department of Cardiology, PNS Shifa Hospital, Karachi, Pakistan; Department of Medicine, Indus Hospital, Lahore, Pakistan; Department of Surgery, Indus Hospital, Lahore, Pakistan; Department of Medicine, Royal College of Surgeons in Ireland, Dublin, Ireland; Weill Cornell Medical College- Qatar, Doha, Qatar; Department of Medicine, Royal College of Surgeons in Ireland, Dublin, Ireland; Cardiovascular Analytics Group, Hong Kong, China; Department of Medicine, Royal College of Surgeons in Ireland, Dublin, Ireland

## Abstract

Fasting is a part of many world religions and in Islam fasting is obligatory for every adult Muslim during the month of Ramadan. Islam has exempted sick people from fasting; however, many people still partake in this activity. We investigated how Islamic fasting affects patients with heart failure with preserved ejection fraction (HFpEF). We enrolled 938 patients (fasting n = 456; non-fasting = 482) in this prospective observational study. The fasting group showed a decrease in NYHA functional class III (23.36% vs. 17.77%; p-value < 0.05) and IV (3.76% vs. 2.19%; p-value < 0.05), and an increase in class I(35.57% vs. 43.64%; p-value < 0.05). symptoms. This is an important area for physicians to advise patients with HFpEF to fast in the month of Ramadan as it can have a favorable effect on their symptoms and quality of life.

## 1 Introduction

Many world religions advocate periods of fasting, and in Islam fasting is obligatory for every adult Muslim during the month of Ramadan. Muslims are to abstain from taking anything per-oral or intranasally from dawn till dusk, a period which varies widely with geographical disparity and the season. Apart from food, medications and other intravenous nutrients are prohibited in Ramadan. Furthermore, there is an alteration of the routine sleep-wake cycle that can cause daytime malaise [1]. Although Islam exempts the sick and the unable from fasting, many Muslims partake in this activity during the religious month. Therefore, it is important to investigate how these changes affect patients with heart failure with preserved ejection fraction (HFpEF). Previous studies have investigated patients with decompensated heart failure with reduced ejection fraction (HFrEF); however, the subset of HFpEF has largely been under-evaluated [2,3].

## 2 Methods

In this prospective analysis, patients with the diagnosis of HFpEF and regular clinical follow-up were enrolled in this study from March to April 2022. Those who would fast in Ramadan were labeled **Group 1** and those who would not as **Group 2**. All patients provided written, informed consent to participate in the study according to the World Medical Declaration of Helsinki. The basic demographic data (with anonymization of personal information), comorbid conditions, history of coronary artery disease (CAD), previous revascularization procedures, current medicines, and HF symptoms based on New York Heart Association (NYHA) functional class, during Ramadan fasting were recorded by the investigators. In addition, we also obtained the etiology of HF and previous history of cardiac implantable electronic device placement. All patients had their baseline hematology and biochemistry performed along with B-type natriuretic peptide (BNP), Troponin I, and lipid profile. In addition, an electrocardiogram (ECG) was performed to assess the heart rhythm and transthoracic echocardiography (TTE) was done to assess baseline ejection fraction (EF), left ventricular chamber size, the grade of diastolic dysfunction, valvular heart disease, and pulmonary artery systolic pressure. A follow-up of the clinical data was performed at the end of April by a repeat interview, laboratory tests, ECG, and TTE. The interview was conducted by a trained physician (W.A.) who obtained the following information: change in NYHA functional class, any change in HF symptoms, diet and medication compliance, hospitalizations, or emergency visits, and MACE.

HFpEF was defined as per the European Society of Cardiology 2021 HF guidelines: (i) symptoms ± signs of HF, (ii) EF ≥ 50%, (iii) objective evidence of cardiac and structural abnormalities consistent with left ventricular (LV) diastolic dysfunction/raised LV filling pressures, including raised BNP [4]. Medication non-compliance was defined as < 80% of HF pills taken for more than 7 days. Non-compliance to diet was defined as non-adherence to fluid and salt restriction as prescribed by the primary physician for more than 7 days. MACE included a composite of total death, myocardial infarction, stroke, hospitalization, and revascularization, including percutaneous coronary intervention. All data were extracted onto the statistical analysis software: Statistical Package for Social Sciences (SPSS) version 26 (IBM Corp., Armonk, NY.). Categorical variables were expressed as percentages (%), and continuous variables as mean ± standard deviation (SD). A Chi-square test was used to analyze categorical variables and Student's t-test and Mann-Whitney test were used for normal and abnormal distribution of continuous variables, respectively. A two-tailed p-value of <0.05 was considered significant.

## 3 Results

We enrolled 1,354 HFpEF patients in this study but 416 were excluded due to poor follow-up and denied consent. After exclusion, 938 patients were included (fasting n = 456; non-fasting = 482). Baseline demographics with pre-and post-Ramadan characteristics are tabulated in Table 1. The fasting group showed a decrease in NYHA functional class III (23.36% vs. 17.77%; p-value < 0.05) and IV (3.76% vs. 2.19%; p-value < 0.05), and an increase in class I.(35.57% vs. 43.64%; p-value < 0.05). Similarly, there was a decrease in diastolic dysfunction grade III/IV (3.49% vs. 2.63%; p-value < 0.05) and improvement to grade I (65.81% vs. 71.27%; p-value < 0.001). However, there was no difference in NYHA class and diastolic dysfunction among non-fasting individuals. Diet adherence and drug compliance were similar between fasting and non-fasting groups but diet adherence increased during the month of Ramadan in the fasting group (86.11% vs. 90.78%; p-value < 0.05). In laboratory parameters, high-density lipoprotein (HDL: 32 ± 3 vs. 44 ± 3; p-value < 0.05), B-type natriuretic peptide (BNP: 139 ± 64 vs. 110 ± 42; p-value < 0.05), and triiodothyronine (T3: 1.7 ± 0.9 vs. 2.4 ± 1.2; p-value < 0.05) improved post-Ramadan in the fasting group.

**Table 1:** Clinical characteristics.

Variable	Pre-Ramadan			Post-Ramadan		
	Fasting (n = 456)	Non-fasting (n = 482)	P-value	Fasting	Non-fasting	P-value
**Age**	56 ± 9	55 ± 11	0.925	–	–	–
**Female**	28.07%	26.97%	0.811	–	–	–
**Comorbidities/etiology**						
Diabetes Mellitus	24.56%	24.89%	0.978	–	–	–
Hypertension	30.92%	29.87%	0.846	–	–	–
Current smoking	19.54%	18.67%	0.815	–	–	–
Chronic kidney disease	5.26%	5.8%	0.834	–	–	–
Hyperlipidemia	13.15%	12.44%	0.795	–	–	–
Asthma/Chronic obstructive lung disease	6.57%	6.22%	0.991	–	–	–
Thyroid disease	7.67%	7.88%	0.802	–	–	–
Peripheral arterial disease	7.01%	6.43%	0.526	–	–	–
Atrial Fibrillation	9.86%	9.33%	0.884	–	–	–
Sleep apnea	2.11%	2.9%	0.819	–	–	–
Coronary artery disease	41.79%	41.49%	0.935	–	–	–
Valvular heart disease	26.31%	26.97%	0.793	–	–	–
Pericardial disease	15.35%	15.56%	0.975	–	–	–
**Prior revascularization**	30.7%	35.26%	**0.015**	–	–	–
**New York Heart Classification**						
I^a^	35.57%	36.73%	0.735	43.64%	37.34%	**<0.001**
II	36.81%	36.12%	0.899	36.4%	36.72%	0.806
III^a^	23.86%	21.23%	0.245	17.77%	20.76%	**0.023**
IV^a^	3.76%	5.92%	**0.026**	2.19%	5.18%	**<0.001**
**Left ventricular ejection fraction**	55 ± 4	54 ± 4	0.912	53 ± 5	55 ± 3	0.846
**Diastolic dysfunction**						
I^a^	65.81%	66.14%	0.766	71.27%	66.39%	**<0.001**
II	30.7%	27.89%	0.082	26.1%	26.76%	0.703
III/IV^a^	3.49%	5.97%	0.053	2.63%	6.85%	**<0.001**
**Drug compliance**	93.15%	94.06%	0.750	93.78%	93.91%	0.872
**Diet adherence**^a^	86.11%	88.63%	0.154	90.78%	91.2%	0.321
**Laboratory tests**						
LDL-C (mg/dL)	124 ± 26	122 ± 32	0.762	121 ± 12	122 ± 34	0.665
HDL (mg/dL)^a^	32 ± 3	34 ± 5	0.432	44 ± 3	35 ± 4	**0.002**
ALT (IU/L)	29 ± 11	29 ± 10	0.995	32 ± 7	31 ± 6	0.814
Hb (g/dL)	11.7 ± 2.6	12.3 ± 1.9	0.075	11.5 ± 2.5	11.9 ± 2.1	0.808
T3 (nmol/L)^a^	1.7 ± 0.9	2.1 ± 0.9	0.057	2.4 ± 1.2	2.2 ± 1.1	0.087
T4 (mcg/dL)	7.7 ± 1.4	7.4 ± 2.1	0.112	7.5 ± 1.3	7.4 ± 2.5	0.204
TSH (mIU/L)	3.4 ± 1.2	3.6 ± 0.9	0.370	3.1 ± 0.8	3.3 ± 0.9	0.211
Creatinine (mg/dL)	1.3 ± 0.6	1.3 ± 0.8	0.992	1.2 ± 0.3	1.3 ± 0.7	0.914
Potassium (mmol/L)^a^	3.6 ± 1.7	4.1 ± 0.9	**0.043**	3 ± 1.2	3.4 ± 1.1	**0.035**
BNP (pg/mL)^a^	139 ± 64	141 ± 50	0.134	110 ± 42	124 ± 46	**0.024**
Trop I (ng/mL)	0.03 ± 0.02	0.04 ± 0.02	0.956	0.04 ± 0.02	0.04 ± 0.01	0.968

a p-value <0.05 between fasting pre-Ramadan and post-Ramadan groups.

## 4 Discussion

Ramadan fasting significantly affects the patient's lifestyle due to the reduced frequency of meals and subsequent consumption of the larger amount of calories at night. Notably, it is difficult to maintain more than 12 hours dosing intervals for several patient groups, including ischemic heart disease, hypertension, and lung diseases. Many studies have investigated the relationship between Ramadan fasting and cardiovascular (CV) diseases. A meta-analysis reported a positive effect of Ramadan fasting on CV risk factors [5]. Two other studies showed improved volume status in fasting patients, favorable effects on atrial fibrillation and lipid profile, and no effects on immediate or long-term outcomes [2,3]. There were several limitations, including the possibility of unmeasured confounding biases due to the observational nature of the study. However, our study represents the first and largest evaluation of the relation between fasting and HFpEF in South Asia.

## 5 Conclusion

The main clinical implication of this study is to advise cardiology physicians to advise patients with HFpEF to fast in the month of Ramadan as it can have a favorable effect on their symptoms and quality of life.

## Please state any sources of funding for your research

NA

## Ethical Approval

NA

## Consent

Written informed consent was obtained from the patient for publication of this case report and accompanying images. A copy of the written consent is available for review by the Editor-in-Chief of this journal on request.

## Author contribution

SA, SH: conceived the idea, designed the study, and drafted the manuscript.

JA, MHR : conducted comprehensive literature search, screened the studies for relevant content, and created the literature review table.

WAR, KSOA: revised the manuscript critically and refined the literature review table.

JM: drafted the discussion part of the manuscript, revised the final version of the manuscript critically based on the reviewer and editorial comments

TA, AKA, RAM: Conceived the initial study idea, conducted the analysis, and gave the final approval for publication.

## Registration of Research Studies

Name of the registry: NA

Unique Identifying number or registration ID: NA

Hyperlink to your specific registration (must be publicly accessible and will be checked): NA

## Guarantor

Talal Almas

RCSI University of Medicine and Health Sciences

123 St. Stephen’s Green

Dublin 2, Ireland

Talalalmas.almas@gmail.com

## Declaration of competing interest

NA
